# Photo-crosslinked lignin/PAN electrospun separator for safe lithium-ion batteries

**DOI:** 10.1038/s41598-022-23038-7

**Published:** 2022-10-31

**Authors:** Yerkezhan Yerkinbekova, Sandugash Kalybekkyzy, Nurbol Tolganbek, Memet Vezir Kahraman, Zhumabay Bakenov, Almagul Mentbayeva

**Affiliations:** 1 National Laboratory Astana, Nazarbayev University, Nur-Sultan, Kazakhstan; 2 Department of Chemical and Materials Engineering, School of Engineering and Digital Sciences, Nazarbayev University, Nur-Sultan, Kazakhstan; 3 Department of Chemistry, Marmara University, Istanbul, Turkey

**Keywords:** Chemical engineering, Materials for energy and catalysis

## Abstract

A novel crosslinked electrospun nanofibrous membrane with maleated lignin (ML) and poly(acrylonitrile) (PAN) is presented as a separator for lithium-ion batteries (LIBs). Alkali lignin was treated with an esterification agent of maleic anhydride, resulting in a substantial hydroxyl group conversion to enhance the reactivity and mechanical properties of the final nanofiber membranes. The maleated lignin (ML) was subsequently mixed with UV-curable formulations (up to 30% wt) containing polyethylene glycol diacrylate (PEGDA), hydrolyzed 3-(Trimethoxysilyl)propyl methacrylate (HMEMO) as crosslinkers, and poly(acrylonitrile) (PAN) as a precursor polymer. UV-electrospinning was used to fabricate PAN/ML/HMEMO/PEGDA (PMHP) crosslinked membranes. PMHP membranes made of electrospun nanofibers feature a three-dimensional (3D) porous structure with interconnected voids between the fibers. The mechanical strength of PMHP membranes with a thickness of 25 µm was enhanced by the variation of the cross-linkable formulations. The cell assembled with PMHP2 membrane (20 wt% of ML) showed the maximum ionic conductivity value of 2.79*10^−3^ S cm^−1^, which is significantly higher than that of the same cell with the liquid electrolyte and commercial Celgard 2400 (6.5*10^−4^ S cm^−1^). The enhanced LIB efficiency with PMHP2 membrane can be attributed to its high porosity, which allows better electrolyte uptake and demonstrates higher ionic conductivity. As a result, the cell assembled with LiFePO_4_ cathode, Li metal anode, and PMHP2 membrane had a high initial discharge specific capacity of 147 mAh g^−1^ at 0.1 C and exhibited outstanding rate performance. Also, it effectively limits the formation of Li dendrites over 1000 h. PMHP separators have improved chemical and physical properties, including porosity, thermal, mechanical, and electrochemical characteristics, compared with the commercial ones.

## Introduction

Lithium-ion batteries (LIBs) have been lauded as one of the most promising power sources for portable electronic gadgets and electric vehicles due to their high energy/power density, wide operating voltage range, low self-discharge rate, and long cycle life^1,2^. Current progress on Li-ion batteries mainly focuses on designing novel and high-capacity active materials along with advanced electrolyte materials^3–8^. Yet, it should be taken into consideration that the lithium ion-conducting membrane also has a huge impact on battery performance. Generally, polyolefin-based microporous membranes such as polyethylene (PE) and polypropylene (PP) membranes have been the most used separators in current commercialized LIBs because of their good mechanical, electrochemical stability, and a thermal shutdown features^9,10^. Nevertheless, there have been many reports on challenges of these two materials in terms of low porosity (~ 40%), limited thermal stability, and insufficient electrolyte wettability^11,12^. Thus, it is crucial to fabricate novel membrane materials that overcome all the above-mentioned critical problems. So far, researchers have been recently focusing on a developing alternative separator with higher ionic conductivity produced from sustainable and bio-derived products with enhanced mechanical and thermal stability. Biomaterials such as chitosan, cellulose, algae, fungi, lignin, and others provide a variety of benefits in terms of material availability, environmental friendliness, cost-effectiveness, and, most notably, multifunctionality due to a structural and compositional variety^13–16^. Particularly, lignin is the second most common natural biopolymer after cellulose, which is mainly extracted from wood^17–19^. Xie et al.^20^ presented a study, which was focused on improving the electrolyte-immersed cellulose/lignin separator's mechanical properties for LIBs. Furthermore, Zhang et al.^21^ have described a straightforward method for employing a lignin nanoparticle (LNP)-coated Celgard (LC) membrane as a Li–S battery separator. The presence of various types of polar groups in lignin enables an excellent affinity for liquid electrolytes making them attractive to develop such membrane materials^22^. So far, only a few studies on lignin-containing membranes have been reported. In addition, the importance of material processing while fabricating such membranes has not been discussed as compared to other technologies. One of such developed membranes is a lignin-PVA separator reported by Uddin et al.^23^. It demonstrated high electrolyte uptake (~ 500%) and electrochemical behavior owing to its porous structure^23^. Still, obtained lignin/PVA membrane was quite thick (~ 90 μm), while not thicker than 25 μm membranes are preferred for LIB separators. Such nanofiber structured membranes have high porosity (60–90%), high surface-to-volume ratio, with interconnected pores between fibers, and excellent flexibility which is an excellent approach for the development of separator materials. The electrospinning is one of the simple and manageable operation processes for fabrication of continuous, durable, nanofiber structured materials^24^. By this technique Lignin-PAN (L-PAN) separators were successfully prepared for LIBs, where the porosity of the L–PANs reached 74%^25^. Due to the high polydispersity index and low spinnability of pure lignin solution, it’s difficult to generate continuous nanofibers by electrospinning. Therefore, various strategies have been applied to electrospun lignin, including blending with synthetic polymers^26^, crosslinking^27^, and chemical modifications^28^. Poly(vinylpyrrolidone) (PVP)^29^, poly(vinyl alcohol) (PVA)^30^, poly(vinylidene fluoride) (PVDF)^31^, and poly(acrylonitrile) (PAN)^32^ are the commonly used polymers for developing electrospun separators. Among them, PAN is the most suitable polymer for nanofibrous separators owing to its remarkable chemical and physical durability with easy processability, oxidative degradation resistance, and electrochemical stability^33–35^. PAN also reduces the formation of lithium dendrites during the charging/discharging processes^36^. The physicochemical and thermal characteristics of lignin (e.g., molar mass, solubility, and softening temperature) can be tuned by attaching upper mentioned polymers through chemical modification to customize the properties of the resultant product for diverse purposes. Particularly, chemical modification of lignin with maleic anhydride forms double bonds in the structure which facilitates crosslinking with other oligomers^37^.

In this study, a dual crosslinked electrospun nanofibrous membrane with maleated lignin (ML) and poly(acrylonitrile) (PAN) is presented as a separator for LIBs. The ML/PAN solution was mixed with polyethylene glycol diacrylate (PEGDA) and hydrolyzed 3-(Trimethoxysilyl)propyl methacrylate (HMEMO) to form nanofibrous separators by UV-electrospinning process. In addition, the mechanical strength of the membrane was enhanced by a thermal sol–gel crosslinking process through Si–OH groups of HMEMO. The produced PAN/ML/HMEMO/PEGDA (PMHP) separators with an average thickness of 25 μm showed improved characteristics such as high porosity and wettability, heat resistance, advanced mechanical and electrochemical properties compared with the commercial separator. The PMHP2 membrane (20 wt% of ML) with liquid electrolyte showed the highest ionic conductivity value of 2.79*10^−3^ S cm^−1^, which is significantly elevated than that of the same cell with the Celgard 2400 (6.5*10^−4^ S cm^−1^), which is commercialized and widely used as a separator for LIB. Assembled half-cell batteries with PMHP2 membrane and LiFePO_4_ cathode demonstrated excellent compactness and revealed a specific discharge capacity of 147 mAh g^−1^ at 0.1 C. Additionally, it effectively limited the formation of Li dendrites over 1000 h of continoius stripping and plating. The high porosity, mechanical stability and enhanced ionic conductivity of the membrane improved the electrochemical performance of the lithium-ion battery.

## Experimental

### Materials and methods

Lignin (alkali, M_w_ = 10,000), maleic anhydride (MA, pH 0.8), polyacrylonitrile (PAN, M_w_ = 150,000), 3-(Trimethoxysilyl)propyl methacrylate (MEMO, ≥ 97%), polyethylene glycol diacrylate (PEGDA, M_n_ = 575), tetrahydrofuran (THF, anhydrous, ≥ 99.9%, inhibitor-free), dichloromethane (DCM, anhydrous, ≥ 99.8%), N, N-Dimethylformamide (DMF, anhydrous, 99.8%), hexane (laboratory Reagent, ≥ 95%), 1-methylimidazole (≥ 99%) as a catalyst, and benzoyl peroxide as a photoinitiator were purchased from Sigma-Aldrich.

Materials, which are used to investigate LIB tests: Li metal foil (> 99.9%) as an anode and 1 M lithium hexafluorophosphate (LiPF_6_) solution in alkyl carbonates, including ethylene carbonate, dimethylcarbonate, and diethylenecarbonate, EC/DMC/DEC = 1:1:1 (v/v/v) as a liquid organic electrolyte. The slurry was prepared to obtain LiFePO_4_ (LFP) cathode by following chemicals: LFP powder as an active material, conductive acetylene black (AB, MTI, Richmond, CA, USA) and a binder poly(vinylidene fluoride) (PVdF, Kynar, HSV900, Richmond, USA) in N-methyl-2-pyrrolidone solvent (NMP, > 99.5% purity, Sigma-Aldrich, Netherlands) with the mass ratio of 8:1:1, respectively. The Celgard 2400 commercial separator with a thickness of 25 μm was provided by Celgard company.

### Separator preparation

#### Synthesis of maleated lignin (ML)

Hajirahimkhan et al.^37^ presented a method for successfully maleating lignin in a prior publication. In a round-bottom flask with vigorous shaking, 2.00 g of lignin was added to 30 mL of tetrahydrofuran (THF). For 1 h, the mixture was agitated to achieve a uniform dark-brown mixture. 1.06 g of Maleic anhydride (MA) and 0.10 mg of catalyst were then added to the previous solution, which was agitated for 2 h at 60 °C in a nitrogen (N2) environment. The dark liquid was then dropped into 100 mL of hexanes while stirring to precipitate a light-brown powder. To eliminate the catalyst and any unreacted chemicals, the powder was dissolved in Dichloromethane CH2Cl2 (20 mL) and rinsed with 60 mL of deionized water (DI). The organic layer was separated in hexane (100 mL) and dried for 1–2 h in a vacuum oven at 60 °C. Maleated lignin (ML) was then obtained and kept in the refrigerator.

#### MEMO pre-hydrolysis

3-(Trimethoxysilyl)propyl methacrylate (MEMO) pre-hydrolysis process was performed according to the technique described in the literature^38^. In a nutshell, 1.2 g boric acid and 5.8 g MEMO were placed in an oil bath after being charged into a dry single-neck round-bottom flask equipped with a magnetic stirred and reflux condenser. The hydrolyzation of MEMO was carried out for 2 h at 75 °C under reflux conditions. The resulting solution includes hydrolyzed MEMO (HMEMO) as well as borate ester (B(OEt)_3_). A volatile borate ester was then vaporized for half an hour under vacuum conditions (400 mbar at 80 °C), yielding an extremely viscous resin.

#### Precursor solution preparation and electrospinning

The PAN/ML/HMEMO/PEGDA (PMHP) with 10, 20, and 30% of ML membranes were fabricated by electrospinning method from the precursor solutions and abbreviated as PMHP1, PMHP2, and PMHP3, respectively (formulations are given in Table 1). First, 1.0 g of PAN was dissolved in 9.0 g of DMF solvent with constant stirring overnight to prepare the 10 wt% of polymer solution. Then HMEMO, PEGDA, and ML were added to the solution with various mass ratios as indicated in Table 1 and stirred overnight. A photoinitiator, benzoyl peroxide, was added to the spinning solution 15 min before the electrospinning process, and the final solution of PMHP was loaded into a 10 ml plastic syringe. A high voltage of 16.7 kV was delivered, and the distance between the needle tip and the Al foil grounded drum collector was 12 cm. UV lamp (Osram ULTRA-VITALUX 300 W, λ_max_ = 365 nm) was used to in situ photopolymerization process at ambient temperature. The flow rate was kept constant at 1.0 mL h^−1^, while the duration of the process was 2 h. The resultant PMHP membranes were dried overnight in a vacuum oven at 60 °C to eliminate the remaining moistures. Finally, the method produces crosslinked PMHP membranes with typical thicknesses of 25 μm.

**Table 1:** The mass ratio of the precursor solutions.

Abbreviation	PMHP1	PMHP2	PMHP3
Membrane formulation	Mass ratio		
PAN solution (10 wt%)	7	6	5
Maleated Lignin	1	2	3
HMEMO	1	1	1
PEGDA	1	1	1
Benzoyl Peroxide (Photoinitiator)	3 wt% of the total mass		

### Performance evaluation

The structure and chemical composition of materials were investigated by using Fourier transform infrared spectroscopy (FT-IR) with the range of infrared region 4000–500 cm^−1^. FT-IR spectra were measured using a Thermo Scientific Nicolet iS10 infrared spectrometer bought in the United States. A field emission scanning electron microscope was used to study the morphology of PMHP membranes, as well as the Celgard 2400 separator (SEM, ZEISS Crossbeam 540, Germany). Since the membranes are electron insulators, gold with a thickness of 5 nm was placed on PMHP surface using an Automatic sputter coater Q150T to increase electron conduction. An absorption method using n-butanol solution was used to define the porosity of the membranes. The nanofibrous membrane was immersed in n-butanol for 2 h. The mass of the nanofibrous membrane was measured before and after soaking. The following equation was used to calculate porosity:

1 $${\text{ Porosity }}\left( {\text{\% }} \right) = \frac{{w_{2} - w_{1} }}{{\rho_{b} V}}$$

where *w*_1_ and *w*_2_ are the weights of the separator before and after the soaking in n-butanol, respectively, $\rho_{b}$ is the density of n-butanol, and *V* is the geometric volume of the membranes.

Electrolyte uptake (EU) was measured for membranes with commercial 1 M lithium hexafluorophosphate (LiPF_6_) electrolyte dissolved in EC/DMC/DEC (organic solvents; 1:1:1). The obtained membranes were soaked in the commercial organic liquid electrolyte LiPF_6_ in an inert atmosphere for 2 h at ambient temperature before being wiped clean. Weights were recorded before and after immersing in liquid electrolyte, and uptakes were estimated using the following formula:

2 $${\text{ EU }}\left( {\text{\% }} \right) = \frac{{w_{4} - w_{3} }}{{w_{3} }} \times 100\%$$

where $w_{3}$ and $w_{4}$ are the weights of the separator before and after the soaking, respectively.

The thermal properties of PMHP and Celgard 2400 membranes were investigated using a Simultaneous Thermal Analyzer (STA) 6000 and a thermal shrinkage tests. For TGA, all separators were heated to 600 °C at a rate of 10 °C/min in a nitrogen atmosphere. Thermal shrinkage of membranes is determined by comparing the area of the separators before and after 15 min of exposure at 150 °C.

The chemical stability of PMHP membranes in the liquid electrolyte was checked by calculating gel fraction values. The gel fraction (GF) was measured by immersing the tested materials (d = 19 mm) in 20 mL organic solvent, Dimethyl Carbonate (DMC) for 24 h at ambient temperature. After carefully removing solvent residue, the sample was dried up overnight in a vacuum oven at 60 °C. The gel fraction is computed using the equation and results were indicated in the Supplementary Information (Table S1):

3 $${\text{GF }}\left( {\text{\% }} \right) = { }\frac{{W_{remained} }}{{W_{total} }} \times 100\% .$$

where $W_{total}$ and $W_{remained}$ are sample weights recorded before and after the test.

Standard tensile stress–strain tests were used to measure the mechanical properties of the obtained PMHP membranes. Typical stress–strain test was carried out on WDW-3 machine (Jinan Kason Testing Equipment CO., LTD.) with displacement speed of 1 mm/min. Tensile testing samples were created by cutting the electrospun nanofiber mats into 5 cm * 10 cm strips. A thickness gauge (caliper) was used to determine the thickness of membranes. All samples had the same thickness of 25 μm. The cut electrospun nanofiber membranes were then mounted in the microtensile tester.

Electrochemical impedance spectroscopy (EIS) was used to measure the ionic conductivities of membranes impregnated with a liquid commercial electrolyte at frequencies ranging from 0.1 Hz to 1 MHz and an AC amplitude of 5 mV. The stainless steel (SS) was employed as a blocking electrode and following equation was used to compute the ionic conductivity:

4 $$\sigma = \frac{d}{{R_{b} *S}}$$

where $d,$ $S$ and $R_{b}$ are membrane thickness, the area of contact between membrane and SS, and the bulk resistance, respectively. The electrochemical stability of PMHP membranes was determined via using linear sweep voltammetry (LSV) test in conjunction with the assembly of a CR2032 type coin cell using Li metal as the reference electrode and SS as the working electrode. The potential voltage range at ambient temperature was from 2.0 to 6.0 V with a scan rate of 0.1 mV s^−1^. Long-term Li plating-stripping cycling was analyzed galvanostatically with a current density of 0.5 mA cm^−2^ to examine the stability of membranes towards very reactive Li metal electrodes. LSV, EIS and galvanostatic Li stripping/plating data were analyzed by VMP-3 potentiostat/galvanostat device.

The electrochemical performance was studied by assembling CR2032 coin cells with Li anode, LFP cathode, and commercial LPF_6_ electrolytes. The cathode was obtained by mixing LFP powder (80%), conductive acetylene black (10%), and binder PVDF (10%) in NMP solution, then casting it on Al foil current collector by doctor blade method and drying overnight at 60 °C. The cycling and C-rate performance were measured using a battery testing equipment (BT-2000, Arbin Instruments Inc., TX, USA) by adjusting the C-rate from 0.5 to 5 C in a voltage window of 2.0–4.2 V.

All measurements were obtained for an average of 5 sample results.

## Results and discussion

**Figure 1:**
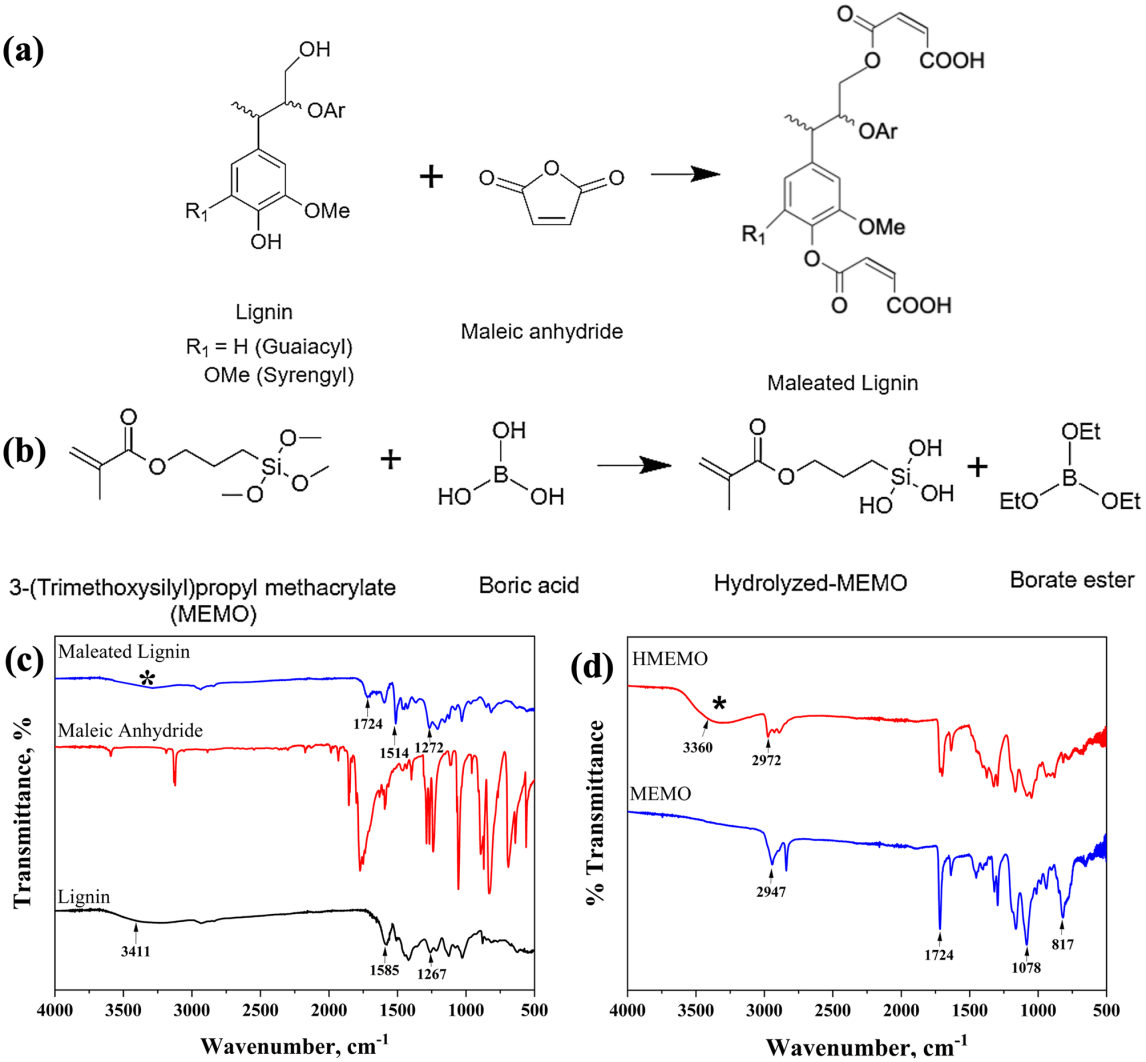
Reaction schemes of (**a**) lignin modification and (**b**) MEMO hydrolysis; FT-IR spectras of (**c**) pure lignin, maleic anhydride, and maleated lignin, (**d**) MEMO and hydrolyzed MEMO.

Prior to fabrication of crosslinked nanofibrous separator, alkali lignin was modified with maleic anhydride. The functionalization of lignin includes the nucleophilic attack on acyl carbon center of the maleic anhydride molecule by an ion pair of the lignin hydroxyl group. Figure 1a shows the esterification reaction that can occur between lignin and maleic anhydride. The maleation process was confirmed by FT-IR analysis. The structural alterations in the functional groups of pure and modified lignin were investigated. The stretching of hydroxyl groups (–OH) in phenolic and aliphatic compounds caused a wide peak at 3411 cm^−1^ in the pure lignin spectra (Fig. 1c). The intensity of this band decreased after the modification procedure, as shown for maleated lignin (ML). The emergence of absorption bands at 1724 cm^−1^ (C=O stretch) was caused by esterification reactions with melic anhydride, which resulted in a reduction in the hydroxyl groups of lignin^39^. This indicates the synthesis of carboxylic acid by swapping lignin and acetyl groups for the hydrogen atoms of the hydroxyl groups. When comparing the spectra of pure lignin with ML it is clear that peaks at 1724 cm^−1^ were the most prominent characteristic peaks in the spectrum of maleated lignin samples^40^. After chemical modification of lignin slightly shifted to the right and left^41^. The bands that indicate a chemical modification in maleated lignin are the region between 1633 and 1217 cm^−1^ (C=C), with the band in 1514 cm^−1^ having the maximum intensity. A rise in this peak’s intensity indicated that modification had successfully occurred.

MEMO was subsequently hydrolyzed to create liquid precursor solutions into a gel by a condensation reaction between its –OH groups and as well as unmodified –OH groups of lignin (Fig. 1b). In the hydrolysis reaction (Fig. 1b), MEMO and boric acid were mixed at a 1:1 molar ratio according to the literature^38^. The hydrolysis was caused by a ligand exchange reaction between the methoxy group (MeO-) of MEMO and the hydroxyl group (–OH) of boric acid. The strong absorption centered at 3360 cm^−1^ in the FT-IR curve (Fig. 1d) is attributed to silanol groups (≡Si–OH) located at 2972 cm^−1^^38^. The presence of this groups in the sample proves that boric acid successfully hydrolyzed MEMO.

**Figure 2:**
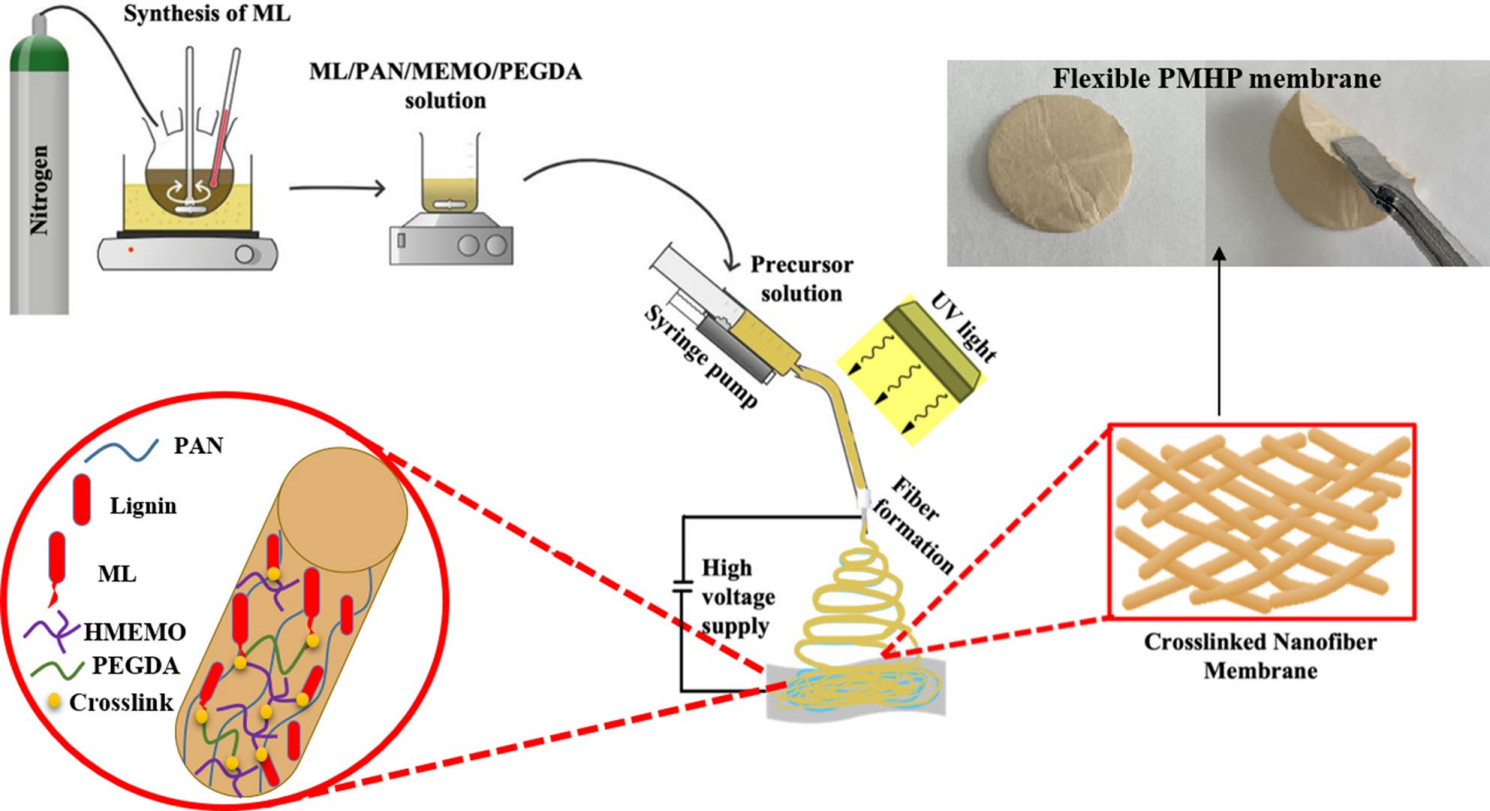
Schematic representation of PMHP membrane via UV-electrospinning technique.

Furthermore, the crosslinked electrospun PMHP membranes were created simultaneously by electrospinning and UV irradiation, as shown schematically in Fig. 2. Ultraviolet (UV) electrospinning is one technique that has gained popularity due to its compatibility with a variety of polymers and ease of incorporation into already-existing electrospinning setups. One of the fundamental goals of this UV electrospinning is crosslinking of monomers during spinning after which more chemically and physically stable fibers can be obtained^42^. Generally, photoinitiators, extra copolymers, or carrier polymers are included in polymer solutions together with photocrosslinkable monomers. Here, UV-crosslinking reaction causes between photosensitive ML, HMEMO, and PEGDA. The ratio of ML, PAN, hydrolyzed MEMO, and PEGDA in an ML-contained membrane was optimized according to Table 1 to fabricate a membrane with high physical, thermal and electrochemical properties with low thickness.

**Figure 3:**
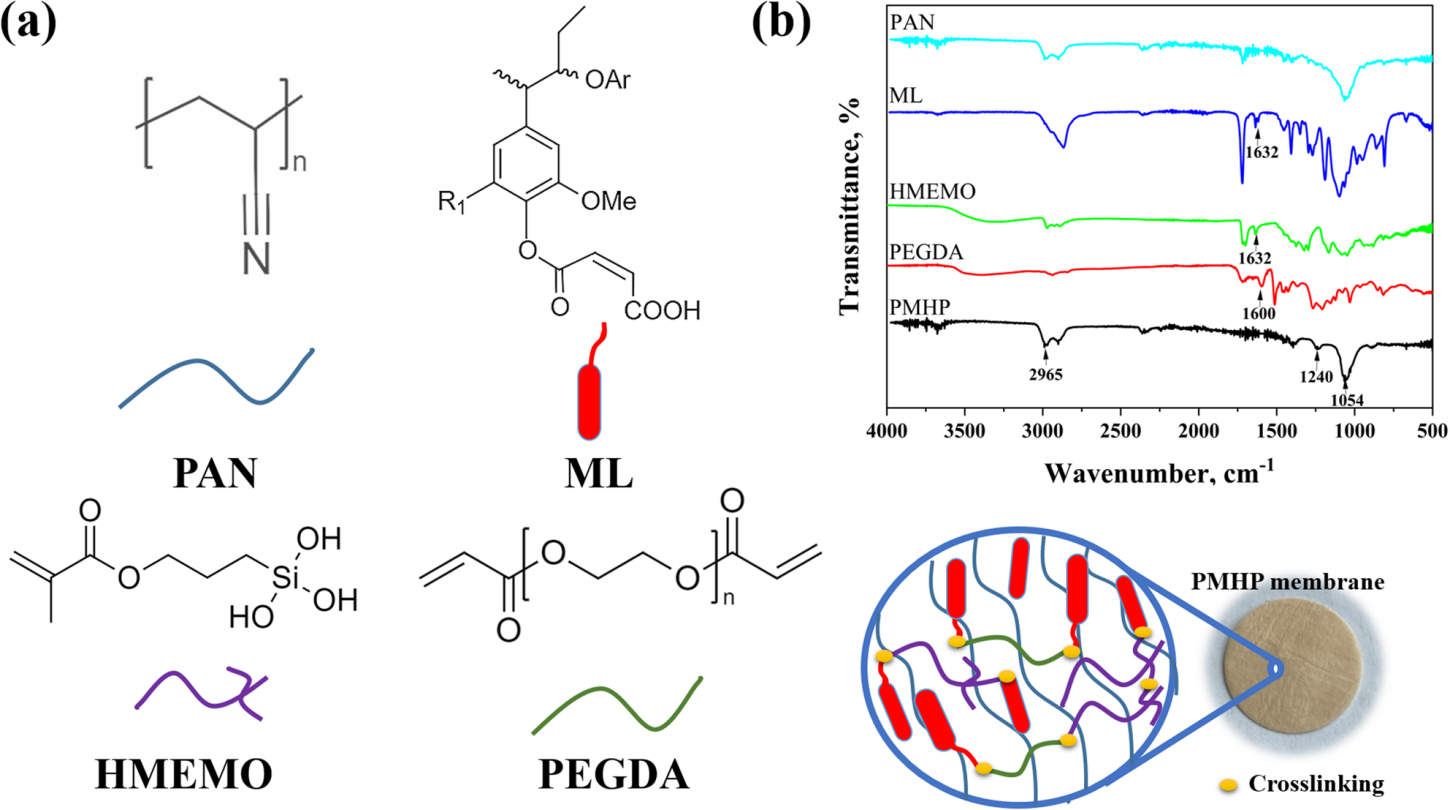
(**a**) Chemical composition, (**b**) FT-IR spectra and photo of PMHP membrane.

Dual crosslinking occurs by both UV-photocrosslinking and sol–gel processes. The acrylic groups of HMEMO and PEGDA and maleic groups of ML are crosslinked during the UV-irradiation. Further, after thermal treatment of the fabricated nanofibers, Si–O–Si covalent bonds are formed with the hydroxyl groups of HMEMO and lignin (Fig. 3). The completeness of the crosslinking reaction in obtained nanofibers were confirmed by leaving the membranes in a solvent for overnight. The result of gel fraction test is given in Table S1. All unreacted and soluble monomers were washed out before applying as a separator. The FT-IR analysis of the obtained PMHP membrane is shown in Fig. 3b. The UV-crosslinking was confirmed by monitoring the FTIR peaks of acrylic C=C (1589–1648 cm^−1^) and maleic double bonds (peaks) after UV-irradiation. Before UV photocrosslinking, the peaks located at 1600 and 1633 cm^−1^ are clearly observed in accordance with the literature^43^, while after the irradiation they disappeared in PMHP spectra, indicating the successful completion of the crosslinking reaction. The peaks near 2970 and 2880 cm^−1^ related to the methyl and methylene groups (–CH and –CH_2_, respectively) in the PAN structure. Also, the nanofibrous membrane exhibit distinctive siloxane bonds (Si–O–Si) stretching signals at roughly 1240 cm^−1^ by condensing the silanol groups of HMEMO during the heat treatment of the membrane. To create the PMHP, it was anticipated that the –OH in the structure of unmodified lignin would create strong contacts with these silanol groups, resulting in the formation of hydrogen and covalent bonds. Such a hybrid structure significantly improves the mechanical strength of the membrane.

**Figure 4:**
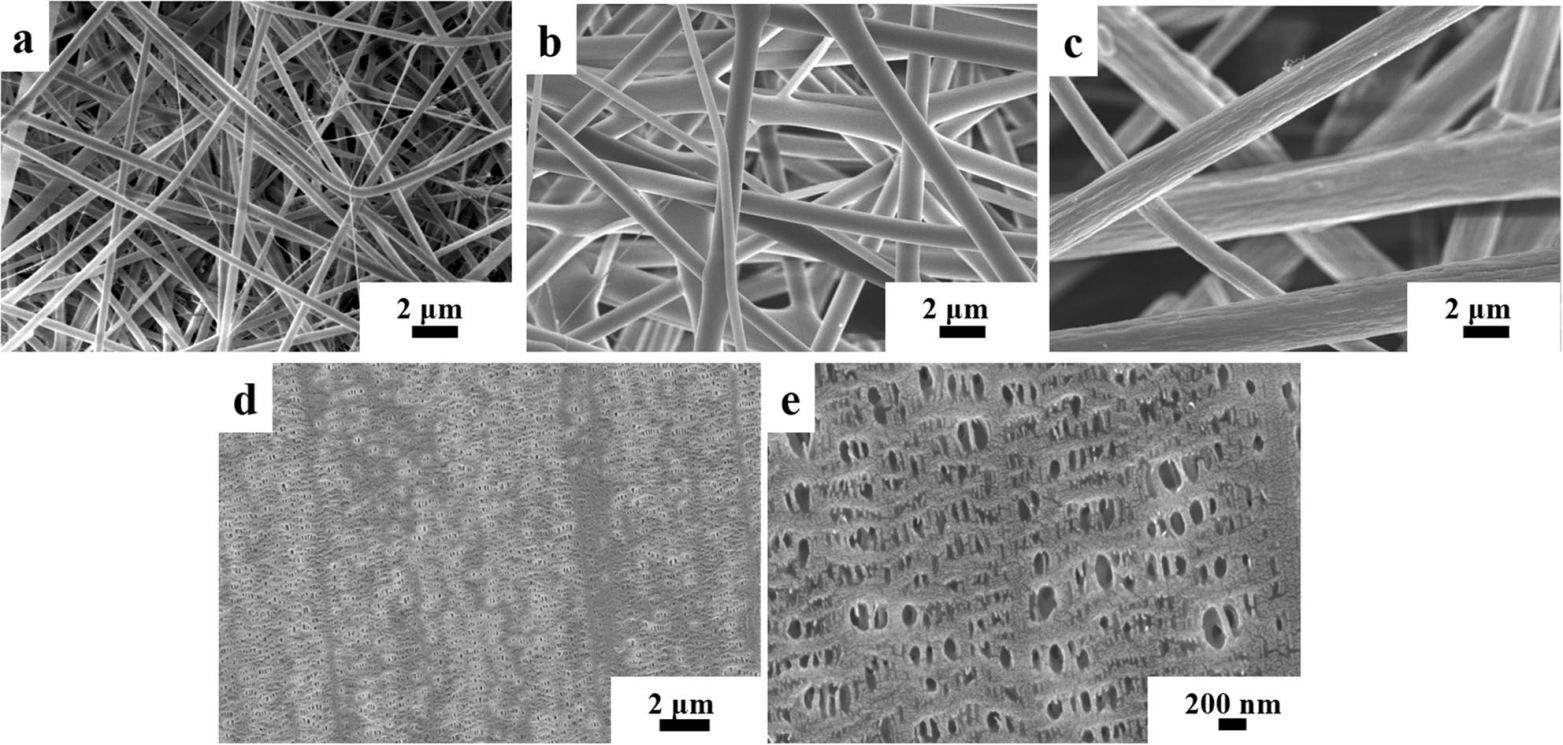
SEM micrographs of PMHP1, PMHP2, PMHP3 (**a**,**b** and **c**, respectively), and Celgard-2400 (**d**,**e**) membranes.

The surface morphology of the electrospun PMHP and commercial Celgard 2400 separators are illustrated in Fig. 4. Figure 4a,b, and c reveal that electrospun PMHP1, PMHP2, and PMHP3 nanofiber membranes are made up of randomly oriented smooth nanofibers without beads and have diameters of about 350, 800, and 1500 nm, respectively. The fibrous membrane contains large voids, and its total porosity is significantly greater than commercial Celgard-2400 membrane. The commercial membrane has a featured microporous structure where pores are dispersed throughout the separator, as shown in Fig. 4d,e. The fiber’s average diameter became greater as the lignin content in the membranes increased, ranging from 350 to 1500 nm (as indicated in Fig. S3). The increase in viscosity caused by the addition of ML is one of the key factors why PMHP membranes with more than 30 wt% ML could not be produced. As seen in Fig. 4c, there is a lot of fluctuation in the fiber sizes in PMHP3, which leads to the PMHP3 membrane exhibiting inferior properties when compared to PMHP2. The rise in diameter revealed that the spinning solution's composition, viscosity, and surface tension had a great impact on fiber size. All the PMHP membranes were found to have a 3D porous network structure with pores linked between the fibers, giving them an ability to absorb liquid electrolytes readily into the membrane structure. This unique structure of PMHP membranes resulted in high porosity, which affects electrolyte uptake and ionic conductivity as well as enhanced mechanical properties.

Porosity can be considered as one of the most critical factors for battery separators, which allows liquid electrolytes to penetrate the membrane and holds more electrolytes, giving an ion migration channel to boost ionic conductivity. Higher porosity promotes EU by enhancing the area of contact between the liquid and the membrane^44^. The n-butanol soak up approach was used to assess the porosity of the membranes and results are listed in Table 2. PMHP2 membrane with a highest porosity (82%) can store more electrolytes and provide more pathways for ion migration. Following that, the porosity of the PMHP3 membrane is reduced to 72%. This is due to the higher difference in fiber sizes in PMHP3, as seen by the SEM images. It has a huge impact on electrolyte uptaking, which is directly impacted by the membrane’s chemical structure and morphology^45^. The increase in diameter demonstrated that the content and viscosity of the solution influenced the fiber size. The porosity of PMHP membranes was significantly higher than that of a commercial Celgard-2400 (41% from manufacturer’s specification) and pure electrospun PAN separators (60%)^36^. This is attributed to the unique fibrous structure of PMHP membranes, which has a high amount of porosity with both open and interconnected voids structures and a large specific surface area that allow for rapid penetration of n-butanol into the membranes. It should be emphasized that the method used to determine porosity can only provide an empirical number that is susceptible to inaccuracies. Another feature, such as employing mercury intrusion porosimetry, might provide more trustworthy data^46^.

**Table 2:** Properties of crosslinked PMHP membrane as a function of ML content and Celgard separator.

Samples/properties	Average fiber diameter (AFD), nm	Porosity, %	Electrolyte uptake, %	The ionic conductivity, S cm^−1^
PMHP1	350 ± 89.1	78.71 ± 6.1	867.35 ± 55.6	1.07*10^−3^ ± 0.09*10^−4^
PMHP2	800 ± 73.1	82.42 ± 7.4	1180.83 ± 42.7	2.79*10^−3^ ± 0.13*10^−4^
PMHP3	1500 ± 291.5	71.53 ± 6.5	822.55 ± 64.6	0.71*10^−3^ ± 0.14*10^−4^
Celgard-2400	–	41**	369 ± 65.7	6.5*10^−4^ ± 0.22*10^−4^

*All measurements were done at room temperature.

** From manufacturer’s specification.

**Figure 5:**
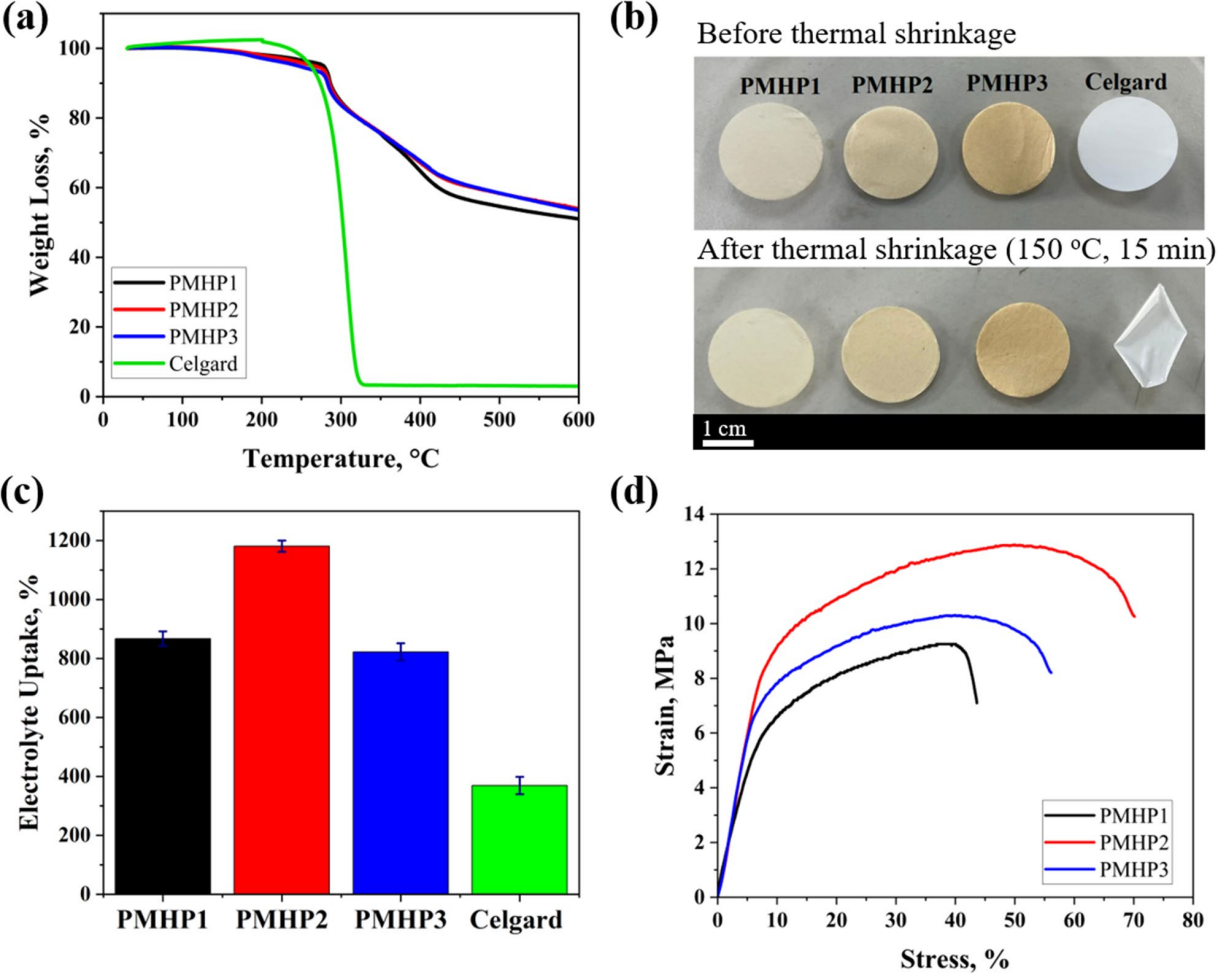
(**a**) Thermo-gravimetric analysis, (**b**) thermal exposure test photos before and after thermal treatment at 150 °C for 15 min, (**c**) liquid electrolyte uptake chart, and (**d**) typical stress–strain curves of PMHP1, PMHP2, PMHP3, and Celgard-2400 membranes.

The electrolyte uptake test is compared in Table 2 and Fig. 5c. EU was measured ten times and the average data was 867, 1180, and 822% for PMHP1, PMHP2, and PMHP3, respectively. The improvement in electrolyte uptake by PMHP membranes was caused mainly by fibrous structure with high porosity and fully interconnected voids, as well as a good affinity for the electrolyte in comparison with the polyolefin separator. High electrolyte uptake by battery membrane hasten the assembling procedure of battery and provides lower resistance for LIBs system^47^.

One of the most significant factors to consider when assessing any storage system safety is thermal stability. A battery separator with excellent thermal dimensional stability is able to increase safety and have a favorable impact on battery performance by preventing the electrodes from contacting each other at high temperatures^48^. Thermal shrinkage of the nanofiber membranes based on PMHP1, PMHP2, PMHP3, and commercial Celgard 2400 separators is measured by comparing the areal change of separators before and after exposure at 150 °C for 15 min to investigate its thermal stability. According to Fig. 5b, the PMHP separators demonstrated excellent thermal and dimensional stability by demonstrating no visible dimensional change and wrinkle. The marketed Celgard 2400 separator, on the other hand, could not keep its original form and shriveled by 40% at 150 °C, and almost decomposed at 200 °C for 15 min (Fig. S2). The UV-crosslinked structure, presence of hybrid Si–O–Si bonds, the durability of PAN polymer, and nanofibrous structure of PMHP membranes improve the thermal property, which is able to successfully prevent battery’s internal short-circuiting and improve its safety at elevated temperatures.

A thermogravimetric analysis (TGA) of the PMHP1, PMHP2, PMHP3, and Celgard separator was undertaken to further investigate the thermal behavior of the membranes. All membranes were heated to 600 °C with a heating rate of 10 °C min^−1^ in a nitrogen atmosphere. According to the TGA thermogram profiles for PMHP membrane (Fig. 5a), the decomposition  begins at 280 °C and completes at 320 °C. A slight weight loss (about 6%) below 170 °C is related to solvent removal, the evaporation of the absorbed water, and several organic constituents suggesting that the separator had relatively minor degradation. The thermal degradation of PMHP happened throughout a temperature range of 250–360 °C because of the complex structure of lignin containing phenolic hydroxyl, carbonyl groups, and benzylic hydroxyl groups. The last weight loss region between 300 and 600 °C is most likely due to polymer backbone degradation. The amount of residue remaining after decomposition at 600 °C is 50 wt%. Heat can be generated during the LIB charge/discharge operation, hence, PMHP separators can withstand high temperatures while preventing the battery from short circuiting.

The separator must be mechanically strong enough to withstand the physical stress induced by external compression and expansion of the electrode materials^49^. The findings of typical tensile stress–strain tests were used to assess the mechanical characteristics of crosslinked nanofiber membrane and are listed in Table 3. PMHP2 membranes have substantially higher Young's modulus values than other membranes, which is expected due to their cross-linked structure^27^. According to the typical stress–strain graph in Fig. 5d, the addition of ML enhanced the tensile strength of PMHP membranes, rising from 9.25 to 12.86 MPa (for PMHP1 and PMHP2, respectively) and dropping to 10.08 MPa for PMHP3 membrane. This is due to the fact that the additional ML is partly connected to HMEMO through condensation between –OH groups in the lignin backbone and HMEMO, as well as the formation of UV-crosslinked bonds.

**Table 3:** Mechanical characteristics of PMHP membranes.

Separators/characteristics	Young’s modulus, MPa	Tensile strength, MPa	Elongation at break, %
PMHP1	129.47 ± 3.91	9.25 ± 0.49	40.31 ± 9.08
PMHP2	183.39 ± 7.82	12.86 ± 1.09	70.12 ± 12.34
PMHP3	169.83 ± 14.08	10.08 ± 1.28	56.05 ± 10.45

Figure 5c shows the results of tensile stress–strain experiments used to assess the mechanical characteristics of crosslinked nanofibrous membranes. The main polymer in the composition of PMHP membranes, PAN, serving as the supporting framework and the 3D cross-linked network structure contribute to the high mechanical strength. It depicts that PMHP2 performed better than other separators and was mechanically stronger to sustain the high stress (~ 12 MPa) during the battery construction process. PMHP3 showed lower mechanical strength due to the high viscosity of precursor solution, and poor surface tension which affected the formation of continuous fibrous structure. All samples demonstrated remarkable mechanical stability, allowing for dry membranes as thin as 25 μm.

**Figure 6:**
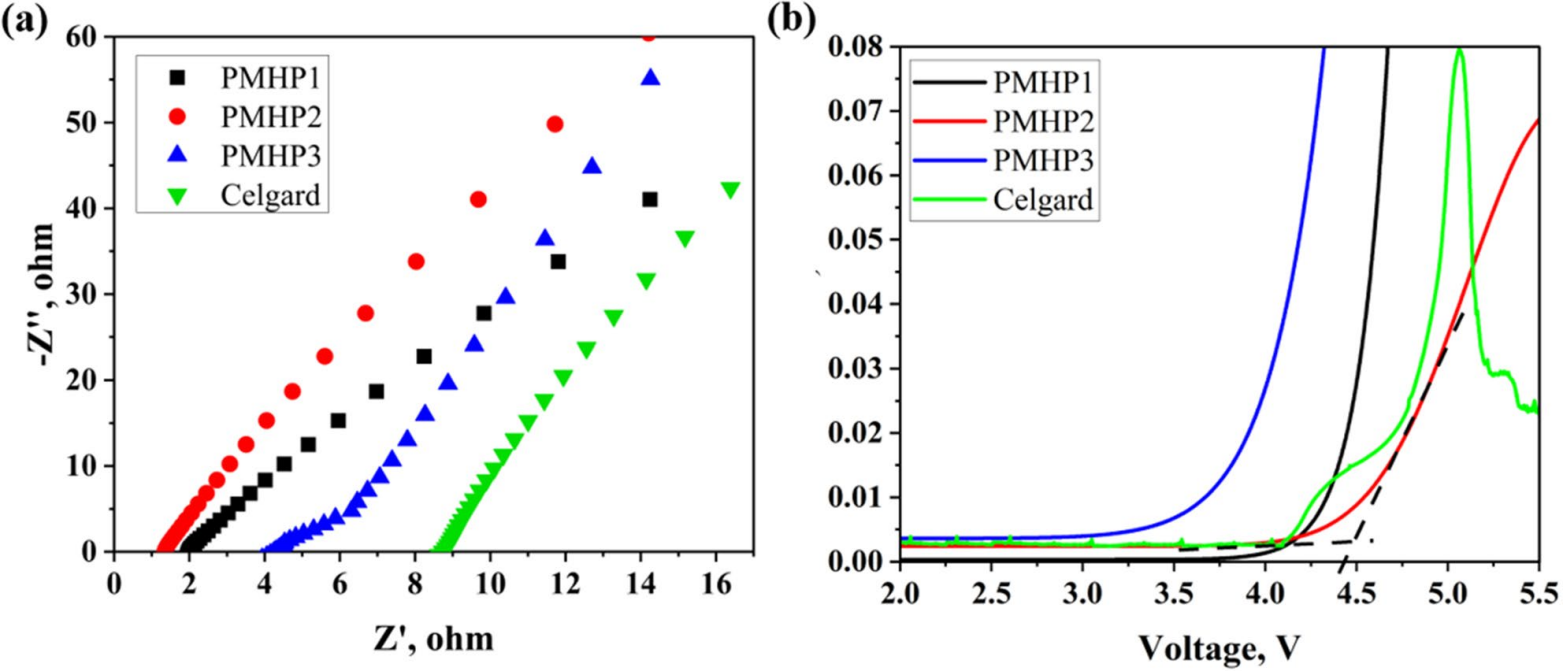
(**a**) Electrochemical impedance spectroscopy (EIS) results of symmetric stainless steel (SS) electrode using PMHP and Celgard 2400 separators; (**b**) Linear sweep voltammogram of Li/ PMHP/SS and Li/Celgard/SS cells.

The fundamental purpose of the membranes for electrolytes is an ion transportation, which is essential for the electrochemical performance of the whole battery. The ionic conductivity of the separator in electrolyte is determined by a variety of factors such as thickness, porosity, structural morphology, and capacity to absorb liquid electrolyte. As a rule, thin separators can increase battery energy density by allowing more area for active materials, while thick membranes cause higher resistance and diminish energy density^50^. All the above-mentioned factors determine the overall effect on ionic conductivity, which were investigated using electrochemical impedance spectroscopy with symmetric blocking electrodes (SS/PMHP/SS). Before constructing the CR2032 coin cells, the thickness of all samples was adjusted to be 25 μm. All membranes had comparable patterns in their impedance spectra (Fig. 6a). Table 2 shows the calculated results of ionic conductivity analysis for PMHP separators. The crosslinking of membrane structure increased conductivity values, which may be ascribed to better porosity and high liquid electrolyte uptake. The highest value of 2.79*10^−3^ S cm^−1^ was observed for PMHP2 separator with 20 wt% of ML content. This result is much higher than that of the commercial membrane of Celgard 2400 (6.5*10^−4^ S cm^−1^) and a pure PAN electrospun separator^51^. For the comparison, in Zhao et al. work, pure PAN and Lignin-PAN membranes (1 : 9, 3 : 7, 5 : 5 by weight) demonstrated the following results: 6.88*10^−4^, 9.94 *10^−4^, 1.24*10^−3^ and 7.75*10^−4^ S cm^−1^, respectively^25^. The increased ionic conductivities in PMHP membranes were ascribed to the nanofibrous structure, which provides a large surface area and fully interconnected porous network, allowing them to absorb significant volumes of liquid electrolyte^44^.

The ability of membranes to have a wide range of potential window stability is crucial in determining their applicability to LIB systems. Linear sweep voltammetry (LSV) was applied to estimate the electrochemical stability of the membranes in a potential range from 2 to 6 V at room temperature with a cell configuration of Li/PMHP/SS and Li/Celgard/SS. Linear sweep voltammograms for PMHP1, PMHP2, and PMHP3 containing cells are presented in Fig. 6b. The cell with PMHP2 membrane exhibits a steady potential window up to 4.65 V, demonstrating that PMHP2 has sufficient electrochemical stability in contrast PMHP1, PMHP3 and Celgard 2400 separators. PMHP3 membrane contained cell demonstrated the lowest voltage stability at 3.5 V, which may be attributed to the degradation of unbounded UV-curable precursors in the composition.

**Figure 7:**
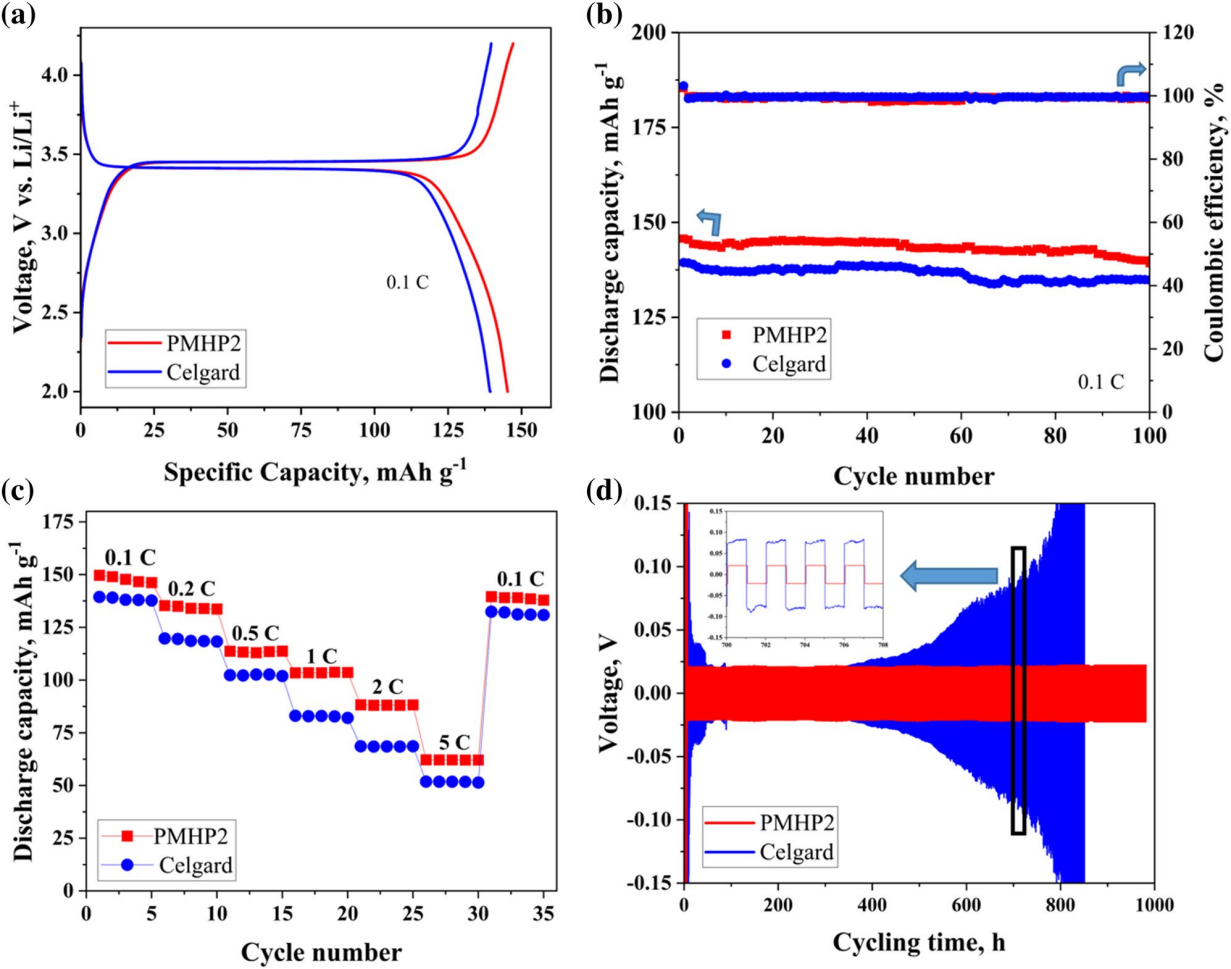
(**a**) Initial charge–discharge, (**b**) Galvanostatic cyclability with Coulombic efficiency at 0.1 C, (**c**) C-rate performance of cells with PMHP2 and Celgard separators with LFP cathode and Li anode, and (**d**) Galvanostatic stripping/plating profiles for Li/PMHP2/Li symmetric cell.

Figure 7a depicts the initial charge–discharge profiles of the cells assembled with the best performing membrane of PMHP2 and commercial Celgard separators at 0.1 C rate. The cells demonstrated potential plateaus of 3.49 and 3.39 V in the charge/discharge processes for the first cycle, which are related to the redox reaction of the LFP cathode^52^. For PMHP2 and Celgard, the cells had initial discharge capacities of 149 and 137 mAh g^−1^ at 0.1 C, respectively. These numbers correspond to about 88% of the theoretical capacity of the LFP (170 mAh g^−1^) for PMHP2 contained cells, while a cell with Celgard membrane exhibited only 80%. Notably, the cells had lower polarization (0.09 V), which is due to the low ion transfer resistance of the functionalized electrospun membrane and advantageous for LIB performance.

Furthermore, after 100 cycles, the cells containing PMHP2 still had a rather high discharge specific capacity of 139 mAh g^−1^ and a capacity retention rate of 93% (Fig. 7b). Higher ion conductivity, which is due to the high porosity of crosslinked nanofiber membrane with interconnected pores, and greater affinity with the commercial LPF_6_ liquid electrolyte, as well as less development of lithium dendrite on membrane surfaces are all factors that contribute to greater performance of PMHP2 compared to the cells with Celgard separator. Coulombic efficiency of 100% proved excellent reversibility of cells and consistent battery cycling performance.

The rate capability of the cells with PMHP2 and Celgard separators was tested with sequentially altering currents between 0.1 and 5 C after 5 cycles and the results are depicted in Fig. 7c. The Li/PMHP2/LFP cell demonstrated outstanding rate capability, where specific discharge capacities were 149, 138, 112, 103, 87, and 65 mAh g^−1^ for 0.1, 0.2, 0.5, 1, 2, and 5 C, respectively. On the other hand, a cell with commercial Celgard 2400 separator appeared to have a much poor capacity at higher currents. After five cycles at high current (5 C) and lowering it to 0.1 C, the cell with PMHP2 still had a significantly higher capacity of around 137 mAh g^−1^. All half-cell studies demonstrate that PMHP improved the electrochemical performance of LIBs proving to be an excellent replacement for the current one.

Furthermore, the PMHP2 membrane should be able to prevent lithium dendrite growth and short circuit. Galvanostatic Li plating/stripping experiments were used to investigate the interfacial stability of the PMHP2 membrane with continuous Li-ion insertion and extraction of a given current density. The symmetric cells with commercial Celgard and PMHP2 membranes serve as a comparison. The potential response of the symmetric cells with the configuration of Li|PMHP2|Li and Li/Celgard/Li was erected in Fig. 7d. The initial overpotential of the battery cell with PMHP2 membrane was between − 150 and 150 mV due to a starting point of lithium dissolution and deposition processes at the beginning that had its impact on the potential response, which in further cycles declined and stabilized at − 20 to 20 mV proving that the kinetic of lithium mobility was balanced^53^. The durability of the membrane was clearly seen from its 1000 h of performing duration with high current density. By comparison with the cells with Celgrad separator which has only 400 h of performing period, followed by a progressive rise in potential. The production of Li dendrites and disruption of the Celgard/Li interface causes an increase in polarization, complicating Li plating and stripping. According to the combination of Chazalviel's^54^ and Monroe and Newman's^55^ models, the ability to restrict Li dendrite formation is attributable to the high lithium-ion transference number and mechanical strength of PMHP2 membranes. Yet, the activation of the PHMP2 membrane requires quite some time, around 5–10 h. Seemingly the initial impedance abruptly decreases which reveals that the membrane needs pre-cycling for constant performance. Furthermore, morphology of the post-cycled PMHP2 nanofiber membrane was studied after cycling for 1000 h using galvanostatic stripping-plating test by disassembling the Li/PMHP2/Li cell and washing the electrolyte by dimethyl carbonate (DMC). The nanofiber membrane's size, shape, and structural integrity are all well preserved, as illustrated in Fig. S1, indicating high structural stability even after 1000 h of cycling.

## Conclusion

UV-electrospinning approach was used to produce a novel functionalized nanofibrous separator for LIBs by adding biopolymer lignin to the PAN-based membrane structure. The material's electrochemical and mechanical characteristics benefit from the structure's design. The membranes have a high degree of porosity, electrolyte uptake, and the crosslinked structure considerably improves the membranes' thermal and mechanical resilience with a thickness of 25 µm. The higher ionic conductivity was found as 2.79*10^−3^ S cm^−1^ for crosslinked electrospun membranes with 20% ML content in the PMHP2 formulation, which is much greater than that of the commercial separator Celgard 2400 (6.5*10^−4^ S cm^−1^). The crosslinked structure and 3D morphology of PMHP2 membrane with 800 nm diameter nanofibers which are tying together to form interconnected porous networks improved the physical characteristics of membranes like porosity, electrolyte uptake, and thermal and mechanical properties. All these factors positively affected the electrochemical performance of the overall cell. The mechanical property, porosity, and ionic conductivity of the membrane were all altered by crosslinking between ML, hydrolyzed-MEMO, and PEGDA, whereas the hydrophilic character of PEGDA's ethoxy chains improved liquid retention capacity. Consequently, the built half-cell battery with PMHP2 membrane had better electrode interface contact and had a high initial discharge specific capacity of 147 mAh g^−1^ at 0.1 C. In addition, unlike the commercial Celgard 2400, the PMHP2 membrane was able to effectively limit the formation of Li dendrites over lengthy 1000 h, which are proved by Li stripping/plating analysis. These findings suggest that the functionalized nanofibrous separator created in this study is a promising one for the LIB system.

## Supplementary Information

Supplementary Information.

## Data Availability

All data generated or analysed during this study are included in this published article and its supplementary information files.
