# Supplementary material for: Photo-crosslinked lignin/PAN electrospun separator for safe lithium-ion batteries
Source: Sci Rep. 2022 Oct 31;12:18272. doi: 10.1038/s41598-022-23038-7 (PMC9622728; doi:10.1038/s41598-022-23038-7)
Supplement: Supplementary file 1 — Supplementary Information. [file 41598_2022_23038_MOESM1_ESM.docx]

**Photo-crosslinked lignin/PAN electrospun separator for safe lithium-ion batteries**

**Supplementary Information**

Yerkezhan Yerkinbekova^1,2^, Sandugash Kalybekkyzy^1,2^*, Nurbol Tolganbek^2^, Memet Vezir Kahraman^3^, Zhumabay Bakenov^1,2^, Almagul Mentbayeva^2**^

^1^ National Laboratory Astana, Nazarbayev University, Nur-Sultan, Kazakhstan

^2^ Department of Chemical and Materials Engineering, School of Engineering and

Digital Sciences, Nazarbayev University, Nur-Sultan, Kazakhstan

^3^ Department of Chemistry, Marmara University, Turkey

Table S1. Gel fraction test results of PMHP membranes.

| **Membrane name** | **GF, %** |
| --- | --- |
| *PMHP1* | 93.4±1.3 |
| *PMHP2* | 98.7±0.9 |
| *PMHP3* | 89.1±2.3 |

As given in Table S1, the chemical stability of PMHP increases from 93.4% to 98.7% for PMHP1 and PMHP2 membranes, respectively, due to the cross-linking of UV-active ML, PEGDA and HMEMO. However, when ML content was 30% (PMHP3) the chemical stability was decreased up to 89.1% due to the uncrosslinked lignin bonds.


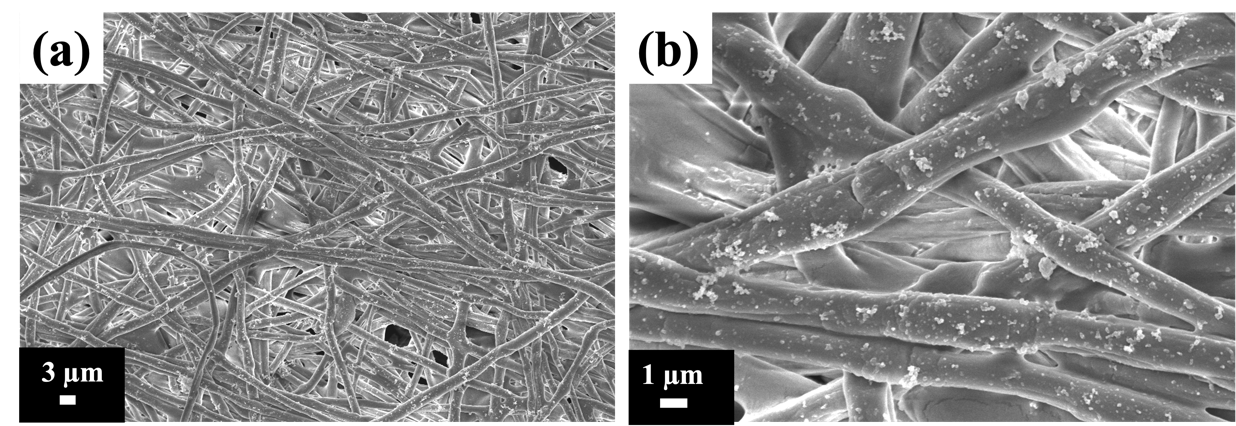


Figure S1. Post-mortem SEM images of the PMHP2 membrane after 1000 hours galvanostatic stripping-plating cycling


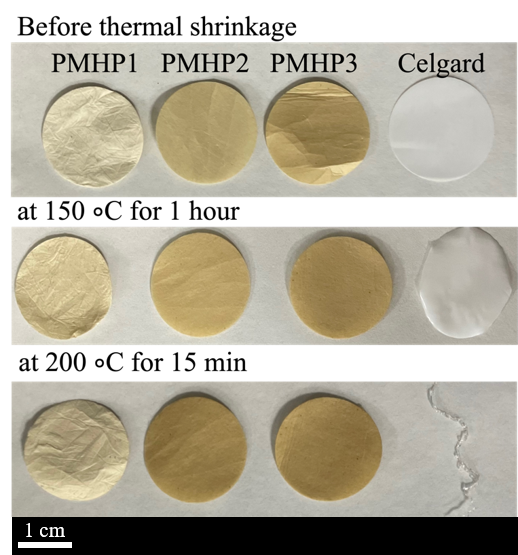


Figure S2. Thermal shrinkage test results of membranes


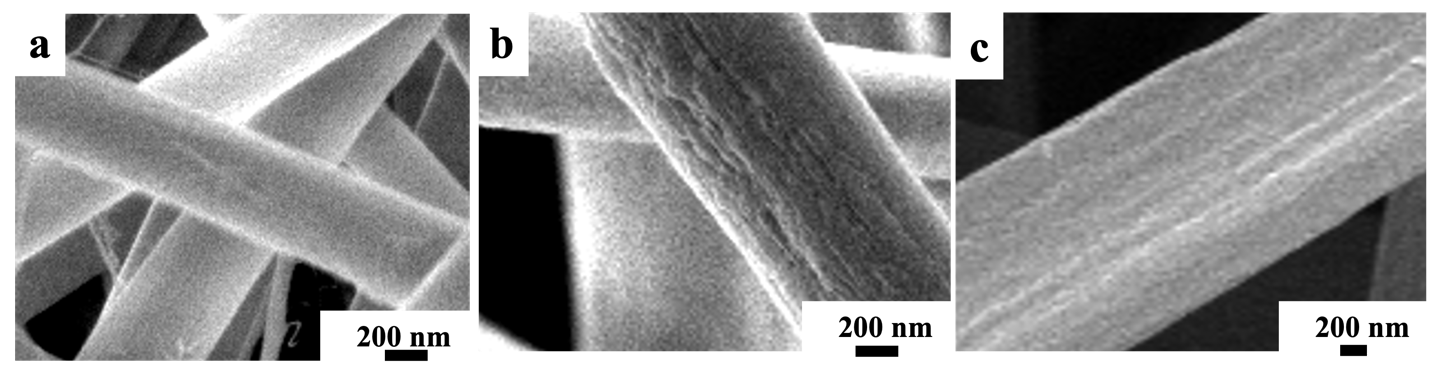


Figure S3. SEM images of electrospun PMHP membranes at higher magnification
